# Exhaustive Exercise Alters Thinking Times in a Tower of London Task in a Time-Dependent Manner

**DOI:** 10.3389/fphys.2016.00694

**Published:** 2017-01-12

**Authors:** Philipp Zimmer, Stephan Binnebößel, Wilhelm Bloch, Sven T. Hübner, Alexander Schenk, Hans-Georg Predel, Peter Wright, Christian Stritt, Max Oberste

**Affiliations:** ^1^Institute of Cardiovascular Research and Sports Medicine, Department of Molecular and Cellular Sport Medicine, German Sport University CologneCologne, Germany; ^2^National Center for Tumor Diseases (NCT) and German Cancer Research CenterHeidelberg, Germany; ^3^Institute of Cardiovascular Research and Sports Medicine, Department for Preventive and Rehabilitative Sport Medicine, German Sport University CologneCologne, Germany; ^4^Chair of Sports Medicine, Chemnitz University of TechnologyChemnitz, Germany

**Keywords:** exercise, physical activity, cognition, executive function, planning, lactate, thinking times, Tower of London

## Abstract

**Purpose:** In contrast to other aspects of executive functions, acute exercise-induced alterations in planning are poorly investigated. While only few studies report improved planning performances after exercise, even less is known about their time course after exhaustive exercise.

**Methods:** One hundred and nineteen healthy adults performed the Tower of London (ToL) task at baseline, followed by a graded exercise test (GXT). Participants were subsequently randomized into one of four groups (immediately, 30, 60, and 90 min after the GXT) to repeat the ToL. Main outcomes of the ToL were planning (number of tasks completed in the minimum number of moves), solutions (correct responses independent of the given number of moves) as well as thinking times (time between presentation of each problem and first action) for tasks with varying difficulty (four-, five,- and six-move problems). Blood lactate levels were analyzed as a potential mediator.

**Results:** No effect of exercise on planning could be detected. In contrast to complex problem conditions, median thinking times deteriorated significantly in the immediately after GXT tested group in less challenging problem conditions (four-move problems: *p* = 0.001, *F* = 5.933, df = 3; five-move problems: *p* = 0.005, *F* = 4.548, df = 3). Decreased lactate elimination rates were associated with impaired median thinking times across all groups ΔMTT4-6 (*p* = 0.001, *r* = −0.309), ΔMTT4 (*p* < 0.001, *r* = −0.367), and ΔMTT5 (*p* = 0.001, *r* = −0.290).

**Conclusion:** These results suggest that planning does not improve within 90 min after exhaustive exercise. In line with previous research, revealing a negative impact of exhaustive exercise on memory and attention, our study extends this knowledge of exercise-induced alterations in cognitive functioning as thinking times as subcomponents of planning are negatively affected immediately after exercise. This is further associated with peripheral lactate levels.

## Introduction

A vast body of literature reports positive effects on cognitive functions of both long-term exercise interventions and single bouts of exercise (Colcombe and Kramer, 2003; Chang Y. K. et al., 2012; Wang et al., 2014). The knowledge of these effects has many implications, including preventive and rehabilitative exercise programs or exercise interventions in different settings such as school or military situations.

One of the most frequently investigated cognitive domains in the context of acute exercise is summarized by the umbrella term “executive functions.” It has become common practice to define executive functioning by enumerating subcomponents such as task flexibility, response inhibition, verbal fluency as well as planning (Burgess, 1997). In contrast to many studies investigating the impact of acute exercise on task flexibility and response inhibition (Barella et al., 2010; Murray and Russoniello, 2012; Hatta et al., 2013; Alves et al., 2014), very few studies focused on planning as a subcomponent of executive functions (Chang et al., 2011a,b; Chang Y. et al., 2012; Hung et al., 2013).

Planning is defined as the ability to identify and select an appropriate sequence of behavior before its final execution and requires the cognitive representation of the starting position, the goal position, and the necessary steps to reach that goal condition (Goel, 2002; Unterrainer and Owen, 2006). The Tower of London task (ToL) is a frequently used and well established assessment to measure planning as a cognitive ability in clinical and non-clinical populations (Keith Berg and Byrd, 2002). Usually, following parameters are assessed in a ToL task: (i) planning, also known as total correct score (number of tasks completed in the minimum number of moves/number of tasks which are perfectly solved), (ii) solutions, also known as total move score (difference between actual moves and minimum number of moves/solved problems independent of the given number of moves), (iii) median thinking times, also known as total initial time (time between presentation of each problem and first action).

To our knowledge, only four studies have investigated planning and planning-associated parameters such as thinking times by using the ToL in the context of exercise (Chang et al., 2011a,b; Chang Y. et al., 2012; Hung et al., 2013). It has been shown that both, acute bouts of aerobic and resistance exercise at moderate to vigorous intensities, increase planning immediately after cessation of the physical interventions (Chang et al., 2011a,b; Chang Y. et al., 2012). Furthermore, Hung et al. (2013) revealed that planning was increased immediately after aerobic exercise, whereas no effects were detected 30 and 60 min post-interventionally. However, thinking time was improved in the exercise group compared to the control group at the 30 and 60 min' post-measurements, indicating a potential sustainable effect of exercise on planning-dependent variables. The previously mentioned studies have two common major methodological limitations. First, they have used questionable sample size calculations and second, a non-computerized version of the ToL was applied, leading to a large investigator-dependent variability in results.

So far, no study was conducted using an exhaustive exercise stimulus. However, recent popular fitness programs, such as high intensity training, freeletics, and Cross-X often include exhaustive exercise stimuli. Since all mentioned studies reported an enhanced ToL performance after a single bout of exercise and have suggested changes in the time course of exercise-evoked influences on planning (Hung et al., 2013), we were interested in the effects of an exhaustive exercise intervention as well as in the sustainability of this potential effect.

In contrast to the distinct opinion that long-term exercise interventions and regular physical activity improve hippocampus-related cognitive functions, such as spatial memory (Rolls, 1991; Wong-Goodrich et al., 2010; Holzschneider et al., 2012; Erickson et al., 2014), studies describing the effects of single bouts/acute exercises on cognition are inconsistent. Positive and negative acute effects of exercise with considerably varying effect sizes have been reported, depending on the exercise regimen (type, intensity, and duration), study population, measured time points, and the assessed cognitive domains (Coco et al., 2009; Chang Y. K. et al., 2012; Perciavalle et al., 2015).

Exercise-evoked increased lactate levels have been hypothesized to be responsible for altered cognitive functions. Coco et al. (2009) and Perciavalle et al. (2015) reported that increased lactate levels, which were induced by an incremental cycling test of maximal exhaustion, were associated with an impaired performance in an attention- and working-memory task 5 min respectively immediately after cessation of exercise. Ten respectively 15 min after completion of the exercise the described effects were not detected anymore. The authors did not only examine the impact of acute exhaustive exercise on following cognitive tasks, but also analyzed the effects of an intravenous and exercise-independent application of a 2 mEq/ml lactate solution which resulted in significantly decreased attention skills. Thus, exhaustive exercise and lactate infusions provided similar results, suggesting that lactate may play a major role in mediating attentional performances or more precisely attentional worsening (Coco et al., 2009). Moreover, Perciavalle et al. (2014) have revealed that submaximal aerobic exercise showed a significant worsening in the participants' attentional performance. Interestingly those effects could only been demonstrated in lactate levels of at least 4 mmol/l. In another study, the authors demonstrate the concurrent rise in blood lactate levels and the number of errors in two specific neurocognitive tests as a marker for the participants' working memory abilities after exhaustive exercise. For both forms of working memory (i.e., non-spatial working memory and motor working memory) they present significant positive correlations between the number of errors and absolute blood lactate levels at the end of the exercise, indicating that lactate might mediate the cognitive impairment (Perciavalle et al., 2015).

Lactate can cross the blood-brain barrier by using monocarboxylate transporters (MCT) to be further metabolized by neurons and glia cells as energy substrate (Bergersen, 2015). Usually, glucose is at least partially metabolized to lactate by astroglia and then allocated to neurons and oligodendrocytes (Bélanger et al., 2011; Jakoby et al., 2014). It has been speculated that an exercise-induced increase in peripheral lactate could utilize that mechanism resulting in a more efficient substrate supply for neurons. Therefore, enhanced cognitive performances after a delay after cessation of exercise seem plausible. However, this concept has been discussed controversially and warrants further investigation.

Furthermore, the research of Coco et al. (2009) and Perciavalle et al. (2015) should be extended by the subcomponent planning. We expected to confirm previous findings of an improvement of cognitive performances following exercise. Considering the time course of this effect, our hypothesis was that exercise would not enhance cognitive performances immediately after the cessation of the GXT, but only at later time points. Moreover, we expected lactate to increase significantly due to the GXT and to potentially mediate the effects of exercise on cognition.

## Methods

### Sample size

Prior to data collection, a power analysis was conducted to determine the particular sample size needed to detect an effect of defined size or larger with a certain probability (statistical test-power: 1-β) as far as this effect does exist in the population. In current research literature, sizes of positive effects of acute exercise on subsequent cognitive performances are reported up to *f* = 0.8 (Hung et al., 2013). A priori power analysis was conducted to determine the sample size needed to detect an interaction effect between time points and the intervention on cognitive performance of *f* = 0.2 with 1-β set at 0.95 and probability of false positive decision and committing a type I error (α), respectively, set at 0.05. Based on results of our recently performed, unpublished pilot studies, correlation of participants' testing scores at t_0_ and at t_1_ was estimated at *r* = 0.50. The power analysis revealed that 112 participants would be required to be distributed evenly on the four groups to achieve the desired 1-β.

### Participants

The study protocol is in accordance with the declaration of Helsinki and was approved by the ethics committee of the German Sport University Cologne. All test subjects gave their written consent before participating.

In order to safely reach the striven sample size and to compensate a possible drop out, 119 participants were included. Subjects were excluded from study participation if they were younger than 18 or older than 35 years of age, had a Body Mass Index (BMI) below 18 or above 30, affirmed any of the questions of the Physical Activity Readiness Questionnaire (PARQ) (Cardinal, 1997) or reported any history of cardiopulmonary, metabolic, neurological or psychiatric diseases. Furthermore, exclusion criteria comprised pregnancy, acute infections, intake of any prescription medication other than oral contraceptives, consumption of illegal drugs during previous months, extensive physical training during the last 2 weeks, <12 h of sleep during the previous 48 h, experience with the applied cognitive tests or any limitation in the ability to exercise or to complete the cognitive testing procedures. Participants were instructed to refrain from exercise and to avoid the consumption of any caffeinated or alcoholic products during the days of their study participation. Participants stating non-compliance to these prerequisites on the day of testing would also be excluded from the study.

### Procedure

Experimental procedures were performed between 8 and 12 o'clock in the morning. After the collection of anthropometric data (sex, age, BMI) and demographic variables (school years, occupational level; Table 1), all individuals executed the baseline testing of the ToL-F (t_0_). Lactate samples and heart rate values were taken directly before and after t_0_. Subsequent to t_0_ the graded exercise test (GXT) was performed. For the second ToL-F testing (t_1_) participants were randomized into one of four groups conducting the test immediately (<3 min), 30, 60, or 90 min after the GXT. During the time from GXT to post-testing, participants sit comfortably and quietly in a waiting room on their own. They were requested to do nothing besides relaxing and recovering. Any activity, including the use of a smartphone or reading, was prohibited. As some participants of an unpublished pilot study suffered from dehydration after exercising, every test subject could drink water *ad libitum*. Lactate samples and heart rate values were assessed preGXT, postGXT, as well as prior to the cognitive assessment at t_1_ (post-test).

**Table 1:** **Characteristics of study participants**.

**Baseline**	**Immediately**	**30 min after**	**60 min after**	**90 min after**	***P***
	***N* = 30**	***N* = 30**	***N* = 30**	***N* = 29**	
Sex	f: 10 (33.3%)	f: 13 (43.3%)	f: 10 (33.3%)	f: 8 (27.6%)	0.725
	m: 20 (66.7%)	m: 17 (56.7%)	m: 20 (66.7%)	m: 21 (72.4%)	
Age	23.33 (3.54)	23.23 (3.41)	23.73 (3.91)	24.48 (4.33)	0.584
BMI	22.70 (2.66)	22.39 (1.98)	22.31 (1.87)	22.98 (2.71)	0.685
Education (school years)	12.73 (0.45)	12.50 (0.51)	12.63 (0.67)	12.72 (0.46)	0.198
Occupational level	Student: 27 (90%)	Student: 27 (90%)	Student: 27 (90%)	Student: 26 (89.6%)	0.660
	PhD student: 1 (3.3%)	PhD student: 2 (6.7%)	PhD student: 0	PhD student: 0	
	Employed: 2 (6.7%)	Employed: 1 (3.3%)	Employed: 3 (10%)	Employed: 3 (10.3%)	
Mean exercise time (min)	24.40 (5.61)	23.60 (4.90)	24.21 (5.43)	24.10 (4.91)	0.943
Fitness (W/kg)	3.60 (0.60)	3.61 (0.55)	3.69 (0.61)	3.57 (0.51)	0.884

*f, female; m, male; BMI, Body mass index; age, BMI, school years, mean exercise time, and fitness are presented as means (±standard deviation). Sex and occupational level are presented as absolute numbers and percentages*.

### Demographic and anthropometric variables

Demographic variables such as age, gender, education level, and current occupation were determined through self-reporting. Education levels were divided into “no high school diploma” (<10 years of school education; coded as 1), “high school diploma after 10 years of school” (coded as 2), “high school diploma after 12 or 13 years of school” (coded as 3), “completed apprenticeship” (coded as 4), “university degree” (coded as 5). Current occupation was structured in “student,” “PhD-student,” and “employed person.”

### Graded exercise test (GXT), fitness level, and lactate

The exercise intervention consisted of a GXT on a stationary cycle ergometer (Lode Excalibur Sport, Groningen, Netherlands), which was also used to determine participants' fitness. Participants were instructed to constantly pedal at 70 revolutions per minute (RPM) beginning at 50 W. The workload was increased every 3 min by 30 W until volitional exhaustion. At the end of each stage heart rate (HR; FT1-Polar heart rate monitor and Wear Link soft-strap transmitter, Polar Electro, Finland) was captured. Moreover, 20 μl of capillary blood were withdrawn from the participants' earlobes to determine whole blood lactate concentration at the beginning as well as at the end of each stage (Biosen S-Line, EKF Diagnostics, Barleben, Germany). True maximal effort was assumed if participants met all three of the following criteria: venous lactic acid concentrations of more than 8 mmol/l, a failure of heart rate to increase with further increments in exercise intensity, and indications of maximal exhaustion (rating of perceived exertion ≥ 17). On the basis of these criteria true maximum effort was assumed for all participants. These and other spirometrical criteria have been recommended by the American College of Sports Medicine (ACSM) in a same or similar manner to confirm maximal effort and have been used in previous studies (Leckie et al., 2014; Pescatello et al., 2014; Tsai et al., 2014). As a measure of physical fitness watts per kilogram of bodyweight (W/kg) were calculated for each participant for the last completed stage of the GXT.

### Tower of london–freiburg version (ToL-F)

The ToL is a well-defined and knowledge-lean task with a definite solution that is commonly used to measure planning in clinical and non-clinical populations (Kaller et al., 2011). ToL consists of a board with three vertical rods, holding three differently colored balls in total. The rods are of different heights and, therefore, can hold different numbers of balls. Three balls can be accommodated at the tallest rod on the left, two balls at the rod in the middle, and one ball at the smallest rod on the right. In the computerized version of the ToL, every task, also called problem, consists of two pictures. On the picture in the upper half of the screen, different ball-rod goal configurations are presented, whereas the lower picture provides an identical wooden board set up in certain ball-rod start configurations. The minimum number of moves in that the task can be solved is presented on the left side next to the start configuration for each problem. Subjects are supposed to transform each start state to match the according goal state with as few moves as possible. Only one ball can be moved at a time, they cannot be dropped next to the rods, and only the uppermost ball on each peg is allowed to move. Furthermore, each problem must be solved within a time limit of 1 min (Hinz et al., 2009). This test controls the influence of the three most important structural problem parameters: goal hierarchy, search depth, and number of optimal paths (for explanations see Kaller et al., 2012). Controlling these parameters ensures increasing difficulty with increasing minimum number of moves in a linear fashion (Kaller et al., 2012).

In the present study, the computerized German standard form of the Freiburger version of the ToL (ToL-F) (Kaller et al., 2014) was applied via the software platform Vienna Test-System (Schuhfried, Vienna, Austria). This application of the ToL-F consists of 27 problems (three 3-move problems and eight 4-, 5-, and 6-move problems each). Problems are presented in ascending order regarding the minimum number of moves. The 3-move problems represent a familiarization phase and were not included in the final analysis in order to prevent bias due to initial differences among participants regarding rule comprehension. Therefore, only 24 problems per participant (eight 4-, 5-, and 6-move problems) are included in the test evaluation and scores. The ToL-F testing software provides the number of problems solved within a minimum number of moves, which is widely accepted as an indicator of the participants' planning performance (Hinz et al., 2009). Besides the number of problems solved within the minimum number of moves (i.e., planning), the number of correctly solved problems (i.e., solutions) as well as the median thinking times (MTT) of 4-, 5-, and 6-move problems are measured separately as well as accumulated and used for further evaluation. In general, longer thinking times are associated with enhanced planning performance since they reflect less impulsive actions (Culbertson and Zillmer, 2005). Less challenging tasks might be more susceptible to impulsive actions. Therefore, breaking down thinking times could add valuable information about the effects of exhaustive exercise.

### Data analysis

Empirical associations between participants' cognitive testing scores at t_0_ and t_1_ were calculated using the Pearson's correlation coefficient. Potential baseline differences between intervention groups in age, BMI, and fitness (W/kg) were investigated using separate one-way analysis of variance (ANOVA). ANOVA assumption of homogenous variances for between-subjects factor levels was tested using the Levene's test. Potential baseline differences between groups regarding education levels were analyzed using Kruskal–Wallis test. Potential baseline differences regarding gender and current occupation were investigated using the separate Fisher's exact tests. To check success of the single bout of exercise, the effect of the intervention on HR and lactate was analyzed using separate 3 (time points) × 4 (groups) mixed ANOVAs (see Section Procedure). Interaction effects between within-subjects factor time point and between-subjects factor delay was further investigated breaking down time point effects into groups (simple effects analysis, SEA). Alpha error accumulation at SEA was controlled using the Bonferroni adjustment. A 2 (time points) × 4 (groups) analysis of covariance (ANCOVA) was conducted to determine a statistically significant difference between the four groups on the ToL performance controlling for age, school years, and fitness level. Again, interaction effects between within-subjects factor time point and between-subjects factor delay was further investigated by SEA. For all inferential statistical analyses mentioned above, the significance level was set at α = 0.05. To explore potential associations between lactate elimination (lactate levels prior to t_1_—lactate levels postGXT) and changes in cognitive performance, Pearson's bivariate correlation coefficients were calculated and tested for significance. In order to avoid alpha-error accumulation, Bonferroni correction was used. Therefore, significance level was set at α = 0.0083 for correlation analysis. All descriptive and inferential statistical analyses were conducted using SPSS 22® (IBM®, Armonk, NY, USA). Test power calculations were conducted using free of charge available statistical test-power computation software G^*^Power 3 (Faul et al., 2007).

## Results

### Characteristics of study participants

One hundred and nineteen participants were recruited. No differences for demographic and anthropometric data were detected. An overview of the participants' characteristics is shown in Table 1.

### Intervention check

**Figure 1:**
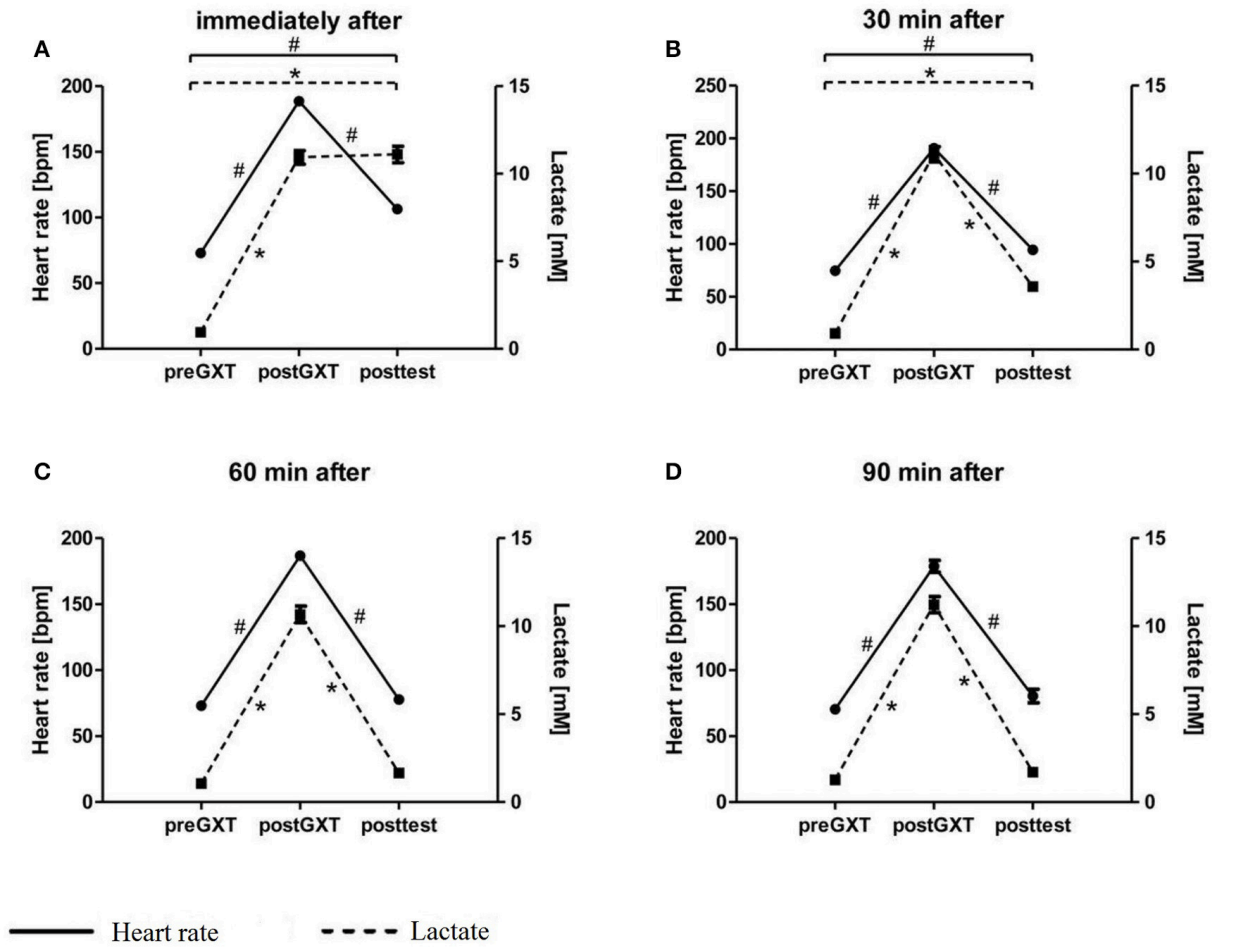
**Intervention check**. Intervention check is presented for each group separately **(A–D)**. Significant changes are pointed out with ^*^ for blood lactate and with ^#^ for heart rate (HR).

A successful intervention can be stated comparing GXT pre-post as well as pre t_1_ (=post-test) values of HR and lactate (Figure 1). Participants reached mean lactate values of 10.98 mmol/l (±2.37) post-interventionally, indicating that the GXT was exhaustive.

### ToL-F results

**Figure 2:**
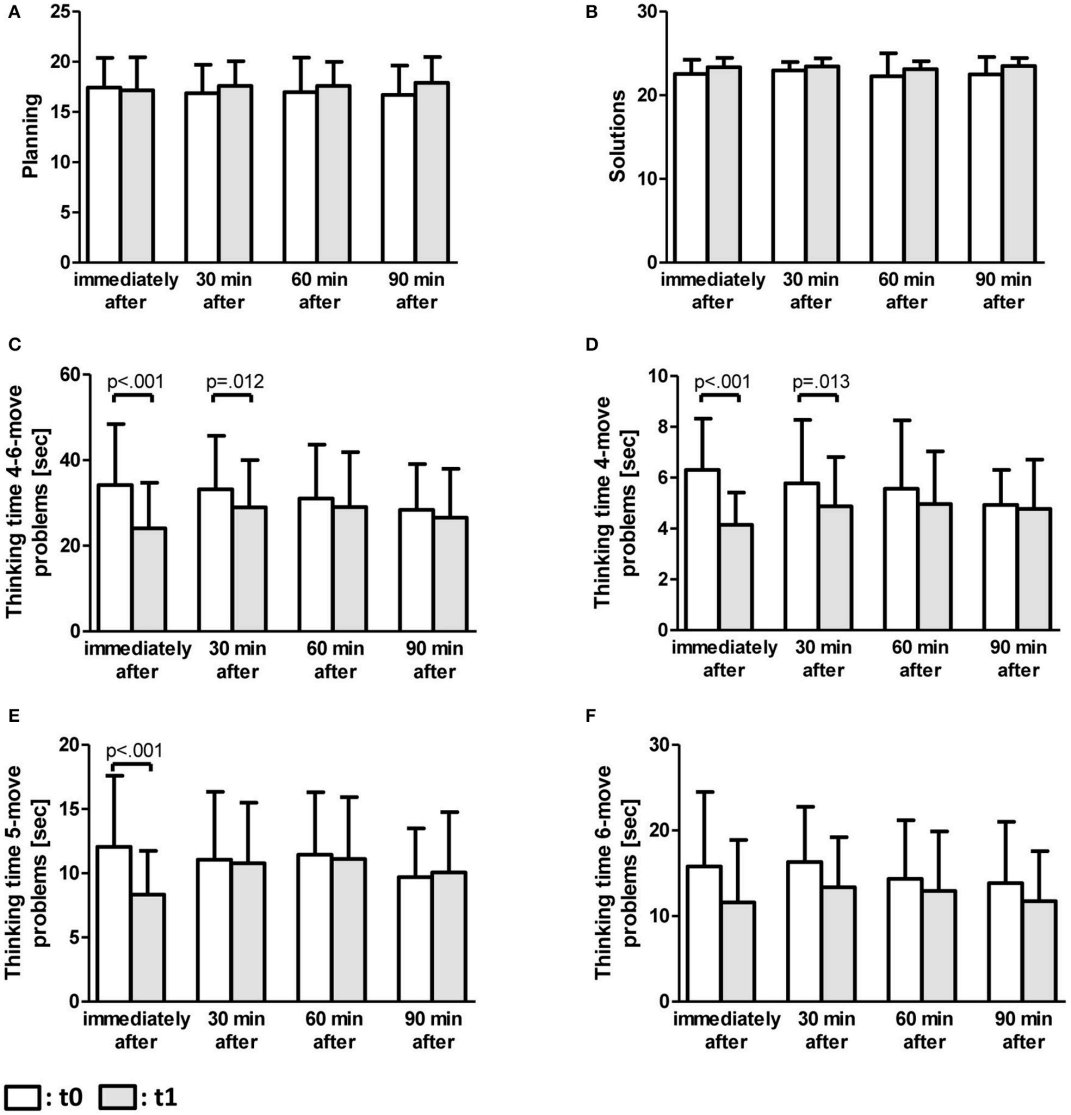
**ToL-F results**. ANCOVA results are presented as means (±standard deviation) for each parameter separately for time points t_0_ and t_1_, controlled for age, school years, and fitness level **(A–F)**. **(A)** Planning, **(B)** Solutions, **(C)** Median thinking time for all 4-6-move problems together, **(D)** Median thinking time for 4-move problems, **(E)** Median thinking time for 5-move problems, **(F)** Median thinking time for 6-move problems. Significant SEA results are pointed out by *p*-values.

In view of planning as the main outcome of the ToL-F, the ANCOVA showed no significant differences for the factors time (*p* = 0.210, *F* = 1.587, df = 1), group (*p* = 0.997, *F* = 0.016, df = 3) as well as for the factor time × group (*p* = 0.178, *F* = 1.666, df = 3; Figure 2A).

Regarding the outcome correct solutions, the ANCOVA revealed no significant differences for the factors time (*p* = 0.866, *F* = 0.029, df = 1), group (*p* = 0.489, *F* = 0.814, df = 3), and time × group (*p* = 0.673, *F* = 0.515, df = 3; Figure 2B).

Median thinking times over all categories of problems (four- to six-movement problems) indicated significant differences for the factor time × group (*p* = 0.009, *F* = 4.063, df = 3), but not for the factors time (*p* = 0.486, *F* = 0.488, df = 1) and group (*p* = 0.641, *F* = 0.563, df = 3). SEA revealed a significant decrease in thinking times for the immediately (*p* < 0.001) and the 30-min group (*p* = 0.012; Figure 2C).

Breaking down the median thinking times into four-, five,- and six-movement problems, the ANCOVA revealed significant time × group differences only for four- and five-movement problems (four-move problems: *p* = 0.001, *F* = 5.933, df = 3; five-move problems: *p* = 0.005, *F* = 4.548, df = 3; six-move problems: *p* = 0.352, *F* = 1.1, df = 3). No significant differences could be detected for the factors time (four-move problems: *p* = 0.315, *F* = 1.018, df = 1; five-move problems: *p* = 0.923, *F* = 0.009, df = 1; six-move problems: *p* = 0.078, *F* = 3.175, df = 1) and group (four-move problems: *p* = 0.714, *F* = 0.455, df = 3; five-move problems: *p* = 0.573, *F* = 0.668, df = 3; six-move problems: *p* = 0.636, *F* = 0.570, df = 3; Figures 2D–F).

For the four-move problems, the SEA showed significant decreases in median thinking times for the immediately (*p* < 0.001) and the 30-min group (*p* = 0.013; Figure 2D). In terms of the five-move problems, SEA indicated significantly reduced thinking times for the immediately tested group (*p* < 0.001; Figure 2E).

### Correlations between Δ LAC and Δ ToL-F results

For all groups accumulated, a significant negative correlation was observed between the lactate elimination rate (lactate prior to t_1_–lactate after GXT) and ΔMTT4-6 (*p* = 0.001, *r* = −0.309), ΔMTT4 (*p* < 0.001, *r* = −0.367), and ΔMTT5 (*p* = 0.001, *r* = −0.290) (Table 2).

**Table 2:** **Correlation analysis of Δ LAC and Δ ToL-F results**.

**Δ LAC (prior to t_1_—postGXT)**		**Δ Planning (t_1_–t_0_)**	**Δ Solutions (t_1_–t_0_)**	**Δ MTT 4-6 (t_1_–t_0_)**	**Δ MTT 4 (t_1_–t_0_)**	**Δ MTT 5 (t_1_–t_0_)**	**Δ MTT 6 (t_1_–t_0_)**
All groups	Pearson's r	−0.117	−0.057	−0.309	−0.367	−0.290	−0.148
	Sig. (2-tailed)	0.206	0.538	0.001^***^	<0.001^***^	0.001^***^	0.112
	*N*	118	118	116	118	118	116

*** *Correlation is significant at the 0.001 level*.

## Discussion

To our knowledge, this is the first study to investigate the influence of a GXT on the time course of planning and its time-related scores as subcomponents of executive functions. In summary, this study gives first hints that planning-associated variables (i.e., thinking times) change within the following 90 min after cessation of exhaustive exercise. The results reveal that median thinking times are significantly shorter immediately and 30 min after completion of the physical intervention. Interestingly, these shifts seem to diminish with increasing time. Negative correlations between the lactate elimination rate and Δ median thinking times could be revealed for all groups accumulated, indicating that higher lactate turnover rates are associated with less impaired cognitive performances. However, the relatively small correlations should not be overestimated and warrant further investigation.

In view of planning, the presented results seem to be contradictory compared to those of Chang et al. (2011b), who reported a significant increase in planning immediately after cessation of a moderate to vigorous bicycle ergometer intervention of 30 min. Hung et al. (2013) stated a positive effect of a single bout of 30 min of moderate ergometer cycling (60–70% heart rate reserve) on planning, namely improved total move scores immediately after exercise, whereas this effect was not detected after 30 and 60 min. However, improvements in correct responses are not limited to the intervention group, but also appear in the control group. This fact suggests that this variable might also be influenced by other factors and confounders. Our results are also inconsistent to those of Hung et al. (2013), who conducted their last assessment 60 min after exercise. Within this time span and even 30 min later (i.e., 90-min group), no significant positive influence of the single bout of exercise on planning could be detected. We want to point out that the sample size calculation of both studies (Chang et al., 2011b; Hung et al., 2013) was based on large effect sizes, which were reported in a study using other cognitive domains as primary endpoints (response inhibition, cognitive flexibility). Furthermore, one study (Chang et al., 2011a) investigated the influence of three different resistance exercise intensities (40, 70, and 100% of the 10 repetition maximum; 10 RM) on the ToL performance compared to a control group. The results showed an increased number of correct responses and a reduction of the total move score immediately after exercise. All other parameters, such as the time-related scores and violation scores did not show any significant differences. In a similar study, Chang Y. et al. (2012) analyzed the effects of two sets of resistance exercises (10 repetitions of 70% of 10-RM of seven exercises) on planning using the ToL. The exercise group showed significant improvements after exercising compared to the pre-tests and significant enhancements when comparing the post-exercise results to those of the control group.

With respect to the total initial time (synonymously titled as the median thinking time in the ToL-F), results of the presented study are partly in line with pre-existing literature. Hung et al. (2013) showed that the total initiation time, as a parameter for response inhibition and the preparation of planning, was extended and thus enhanced 30 and 60 min after cessation of the exercise. It is worth noting that longer initiation times are considered as enhanced planning performances as they represent the establishment of more precise plans and indicate less impulsive actions (Culbertson and Zillmer, 2005). Whereas, prolonged initial times are regularly interpreted as improved cognitive performances in terms of planning abilities (Unterrainer et al., 2004; Asato et al., 2006; Chang Y. et al., 2012), this circumstance is arguable when talking about different executive functions such as inhibition and is commonly interpreted in the exact opposite way. In this case, studies revealed that decreased thinking times do not necessarily lead to worsened inhibition control and that scores remained stable under reduction of thinking or response times (Benikos et al., 2013; Hartmann et al., 2016). Thus, a reduction in response times could also be interpreted as enhanced inhibitory control. As these studies generally use Go/NoGo tasks to evaluate one's cognitive performance and therefore evaluate inhibition processes instead of planning, a proper comparison to our and other studies using the Tower of London task remains limited. Therefore, it seems inappropriate to generalize our results to executive functions other than planning. However, more research is needed to elucidate the role of thinking times in the context of Tower of London tasks. In accordance with the results of Hung et al. (2013), the study of Chang Y. et al. (2012) reveals a significantly prolonged initial time for the exercise condition after the intervention as compared to the pre-test results and the control condition. However, another study of Chang et al. (2011b) only revealed main effects for time and showed that all time-related variables such as the total initial time for both the exercise group and the control group together were significantly shorter in the post-test, indicating an acute deterioration. The latter corresponds with the results of the present study, as we could also demonstrate acute deteriorations in median thinking times of all problem conditions for the immediately after the GXT tested group. These effects are partly detectable in the 30-min group and seem to disappear with increasing time. Because the study of Chang et al. (2011b) does not evaluate follow-up tests, it can only be speculated whether the effect of exercising on time-related scores would decrease similarly as described in the present study. Regarding the median thinking times in dependence of problem difficulty, our results suggest that only less challenging problems (4–5 move problems) are temporally affected. Interestingly, changes in median thinking times for less challenging tasks were further negatively associated with lactate elimination rates.

When comparing the mentioned studies with the present one, some considerations should be kept in mind. First of all, previously performed studies used a different, non-computer- based version of the ToL (Tower of London—Drexel edition; Culbertson and Zillmer, 2005). Using a non-computerized test version involves several limitations. To begin with, time-related scores such as the total initial time are measured by the examiners using a stopwatch. Obviously, these measurements are much less precise as compared to computer-based records and therefore dependent on the experience of the examiner. In addition, time-related scores of the ToL-Drexel edition are totalized across all problem conditions, whereas the ToL-F gives more detailed information by calculating the median thinking times for each problem condition separately (e.g., 4-move problems) as well as accumulated for all problems together. Moreover, the ToL-Drexel edition consists of only ten problems, whereas the ToL-F examines 24 problems, what increases its accuracy. Another critical limitation for a proper comparison between the studies that assessed the impact of exercise on planning and the present study is the fact that strongly varying exercise regimes (type, duration, intensity) were conducted. In addition, one study used a counterbalanced design and did not evaluate pre- and post-exercise tests, what makes an adequate comparison of the results inappropriate (Chang et al., 2011a). Considering the evaluated control groups of the previously described studies, it is worth mentioning that they either watched a video on resistance exercise training (Chang et al., 2011a) or read exercise-related books or articles for a comparable period of time (Chang et al., 2011b; Chang Y. et al., 2012; Hung et al., 2013). Therefore, it cannot be ruled out that those control interventions may have led to cognitive fatigue and thereby pretend larger effects in the end.

Finally, all of the mentioned studies revealed positive effects of exercise on planning. None of them showed significantly improved performances of the control groups, who consistently performed worse as compared to the exercise groups post-interventionally. Partly, the control groups even deteriorated significantly (Chang Y. et al., 2012). Of course, the presented study lacks an adequate control group and the circumstances mentioned above do not replace the missing control group or eliminate the chance of learning effects when repeating the ToL tasks, but they suggest that the changes of planning-associated time-related scores in the present study are due to the single bout of physical exercise and not to learning effects. Other aspects which should be taken into account are learning and motivational processes. However, it appears not plausible that these processes differ significantly between groups and thus cause the described alterations.

An empirical increase in planning can be observed in all groups with exception of the immediately after the GXT tested one. Therefore, one could hypothesize that the improvement in planning after a delay, which may be driven by a learning effect or exercise, is antagonized immediately after cessation of exercise. The significant worsening of the “immediately after” group in terms of median thinking times and the non-significant deterioration in planning of this group could either be explained through an exercise-induced reduction of blood flow in frontal cerebral structures (González-Alonso et al., 2004) or through lactate levels and their exercise-induced fluctuations. The potentially confounding influence of dehydration was controlled through the unrestricted consumption of water. Coco et al. (2009) as well as Perciavalle et al. (2015) showed an inverse correlation of blood lactate levels and cognitive test performances (attention, working memory) and therefore postulate that a decrease in cognitive functions after intensive exercise is mediated by lactate. While we have criticized small sample sizes in the ToL studies previously (e.g., Chang et al., 2011a; Chang Y. et al., 2012), we have to admit that those of the studies investigating lactate as a potential mediator were comparably small (Coco et al., 2009; Perciavalle et al., 2015). Due to differences in lactate production rates, its peripheral metabolism as well as the tissue-specific transporter capacity of individuals, these results should be viewed with caution. As described by Ide et al. (2000) lactate uptake of the CNS is increased during exhausting exercise. In terms of frontal lobe functioning, elevated lactate levels seem to play a crucial, region-specific role. While some studies reported that increased lactate levels are associated with improved excitability in the primary motor cortex (Coco et al., 2010, 2014), other investigations stated contrary results for the frontal cortex as well as for brainstem and spinal cord excitability (Coco et al., 2009, 2011, 2013). Regarding the lactate uptake of the CNS from a biochemical point of view, a strong increase might lead to metabolic irritations through a decrease in pH values, which is driven by the co-transport of protons by the monocarboxylate transporter MCT1 (blood-brain barrier) as well as MCT2 and MCT4 within the CNS (Bergersen, 2015). Though, changes in lactate levels were not associated with changes of planning and solutions, but only with changes in median thinking times. Animal studies provide valuable hints that elevated lactate levels reversibly suppress neuronal firing of hippocampal cells (Gilbert et al., 2006). However, it can be speculated that this inhibiting effect of lactate and protons, resulting in cognitive impairments, converts to an ameliorated energy supply when lactate is metabolized after a delay. This theory is underlined by studies which revealed that glucose uptake by astrocytes is increased after activation, whereas those of neurons remain stable (Bélanger et al., 2011). Since astrocytes' energy supply contributes to only 5–15% of total CNS energy expenditure, it has been hypothesized that an increased energy demand of neurons during activation is served by lactate, which was previously metabolized from glucose in astrocytes (Bélanger et al., 2011; Jakoby et al., 2014). It is well-established that astrocytic lactate can be allocated to neurons by astrocyte-neuron-lactate-shuttles (Pellerin and Magistretti, 1994; Mosienko et al., 2015). Therefore, increased lactate levels after exercise may use this mechanism, leading to an improved metabolic state after a certain delay and thus explain the impacts of exercise-induced increases of lactate on cognition. These considerations would further support findings from Chang's meta-analysis, reporting the largest effects of exercise on cognitive functions after a delay (Chang Y. K. et al., 2012).

This study should be seen within the context of its strengths and limitations. Although, most detected correlations between cognitive performances and lactate alterations are just of weak to medium level, it should be kept in mind that cognitive functioning involves many physiological processes and thus it seems inappropriate to expect much higher correlations. Anyway, the results should not be overemphasized, since correlation coefficients are relatively small concerning the large sample size. Finally, we examined the impact of an exhaustive exercise bout on cognition by testing a demographically homogenous, young, and academic collective of subjects. The assignability to other populations (e.g., non-academic, untrained groups, or clinical populations) is restricted. Probably due to this homogeneity, fitness did not appear as a significant modulator in this study. Anyway, future research could include a more heterogenous study population in order to have a deeper look on the impact of participants' fitness levels on cognitive functioning. Although, the study design based on the assumption that exercise improves planning, the lack of a control group (e.g., one which participates in a stretching program) represents a major limitation and therefore time effects cannot be ruled out. Moreover, further studies might use spirometry testing to get more precise information about the participants' fitness levels and grades of exhaustion. The comparable large sample size and the use of the most objective version of the ToL indicate relatively robust results. In terms of the possible underlying mechanisms by which exercise impacts following cognitive performances, only peripheral lactate levels were measured.

## Conclusion

In view of exercise-induced alterations in planning performances, our study does not confirm previous research. In contrast to that, this study supports previous findings of acute exhaustive exercise-induced deteriorations in cognitive functioning and extends this knowledge by planning-associated thinking times. Our findings suggest that peripheral lactate is associated with thinking times in less challenging tasks after exhaustive exercise. More research is needed to elucidate the effect of exercise on planning and lactate as a potential mediator.

## Author contributions

PZ and SB contributed equally to that work. The other authors helped to design, supervise, analyse, and interpret the study. Moreover, they revised the manuscript critically and gave their final approval. They agree to be accountable for all aspects of the manuscript.

## Ethics statement

Every participant was examined and informed about possible risks before testing. In case that any health issue occurred, the participant was excluded from the study. After reading the information about the study, all participants had the chance to ask questions or to refuse participation. After that all participants gave their written consent.

### Conflict of interest statement

The authors declare that the research was conducted in the absence of any commercial or financial relationships that could be construed as a potential conflict of interest.

## Abbreviations

ACSM: American College of Sports Medicine
ANOVA: Analysis of variance
BDNF: Brain-derived neurotrophic factor
BMI: Body Mass Index
CNS: Central nervous system
GXT: Graded exercise test
HR: Heart rate
LAC: Lactate
MCT: Monocarboxylate transporter
MTT: Median thinking time
PARQ: Physical Activity Readiness Questionnaire
RM: Repetition maximum
RPM: Revolutions per minute
SEA: Simple effects analysis
ToL: Tower of London
ToL-F: Tower of London—Freiburg Version.
